# 630. Spicing Up Safety: A Collaborative Safety Fair and Chili Cook-off to Enhance Burn Awareness

**DOI:** 10.1093/jbcr/irag033.456

**Published:** 2026-04-06

**Authors:** Erin Horrax

**Affiliations:** Legacy Oregon Burn Center, Portland, OR

## Abstract

**Introduction:**

Burn injuries remain a significant public health concern, necessitating comprehensive prevention strategies that engage both healthcare professionals and the community. National Burn Awareness Week provides an opportune moment to implement initiatives that raise awareness and foster collaboration among diverse stakeholders. A community centered Safety Fair & Chili Cook-Off can be used to enhance burn prevention and build lasting partnerships among EMS, burn survivors, and healthcare providers.

**Methods:**

A community focused Safety Fair & Chili Cook-Off was held during National Burn Awareness Week, aiming to foster collaboration among all agencies partnering to care for burn patients in the region and enhance burn prevention education. The event was held at a large event center to accommodate safety vehicles on site for the public to interact with, free parking and admission. Twelve safety teams participated through educational booths, demonstrations and hands on activities for attendees. The chili cook-off featured ten teams comprised of hospital staff, regional fire departments and burn survivors. To further foster relationship building, our judges’ panel included the burn center medical director, EMS medical director, burn survivors and local celebrities to bring continued exposure to the event. Multiple media agencies featured the event and interviewed burn center team members for prevention education. Television, radio and print media were all reached. Over 500 members of the public attended the event. Funds were raised through chili tasting tokens, supporting future burn prevention initiatives.

**Results:**

The collaborative nature of the event strengthened relationships among all those caring for burn patients. The media coverage increased public awareness of burn prevention strategies and National Burn Awareness Week. Funds raised through chili tasting tokens were earmarked for future burn prevention initiatives, ensuring the sustainability of the project's impact.

**Conclusions:**

This initiative exemplifies the power of community collaboration in advancing public health objectives. By leveraging strengths of various stakeholders and engaging the community in meaningful ways; ^2^the event achieved a measurable social return on investment, contributing to a safer and more informed public.

**Applicability of Research to Practice:**

This event exemplifies the principles of community engagement in public health practice. By actively involving diverse stakeholders, it facilitated the cocreation of burn prevention strategies tailored to community specific needs. The positive outcomes observed; ^3^strengthened partnerships, increased public awareness, and funds raised for future prevention initiatives; ^4^underscore the effectiveness of integrating community engagement into prevention. This initiative can provide a replicable framework for integrating community engagement into burn prevention education.

**Funding for the study:**

This event exemplifies the principles of community engagement in public health practice. By actively involving diverse stakeholders, it facilitates the cocreation of burn prevention strategies tailored to community specific needs. The positive outcomes observed underscore the effectiveness of integrating community engagement into prevention. This initiative can provide a replicable framework for integrating the community and partner stakeholders into burn prevention education.

